# Associations of parental reproductive age and elevated blood pressure in offspring: An observational study

**DOI:** 10.3389/fped.2023.990725

**Published:** 2023-03-30

**Authors:** Rui Deng, Ke Lou, Siliang Zhou, Xingxiu Li, Bin Dong, Jun Ma, Jie Hu

**Affiliations:** ^1^ Institute of Child and Adolescent Health, School of Public Health, Peking University Health Science Center, Beijing, China; ^2^ Menzies Health Institute, Griffith University, Nathan, QLD, Australia

**Keywords:** maternal age, paternal age, children, elevated blood pressure, lifestyle

## Abstract

**Background:**

Increased parental reproductive age has been a social trend around the world, and elevated blood pressure in children leads to an approximately two-fold increased risk of hypertension in adulthood. Aim of this study is to assess the associations of parental reproductive age with the risk of elevated blood pressure in offspring, and to explore the influence of offspring lifestyle on the associations.

**Methods:**

Data was obtained from a national school program conducted in 7 Chinese provinces, and the final sample was 39,190 students aged 7–18 years. Anthropometric measurements and questionnaires were designed to collect data of children blood pressure and information respectively.

**Results:**

In this study, 26.7% of children were defined as elevated blood pressure. A U-shaped pattern was observed in the relationship between maternal age and risk of elevated blood pressure, while risk of elevated blood pressure decreased continuously with paternal age increased. After adjustment, offspring of paternal age ≤27 & maternal age ≤26 years and those of paternal age >30 & maternal age >32 years were related to great risk of elevated blood pressure (OR = 1.18, 95% CI: 1.08–1.29, *P *< 0.001; OR = 1.18, 95% CI: 1.01–1.38, *P *< 0.05). When stratified by lifestyle status, significant associations between maternal/paternal age and risk of elevated blood pressure were only observed in those with worse lifestyle behaviors, but not in offspring with healthier lifestyle.

**Conclusion:**

Our findings demonstrate that risk of elevated blood pressure in children is independently related to parental reproductive age, and children maintaining a healthy lifestyle may mitigate the adverse effect.

## Introduction

Increased parental reproductive age has been a social trend around the world. Attention has been paid to the increase in maternal age, whereas an upwards shift in paternal age is also taking place. In the United States, the proportion of first birth to women aged 30–34 years rose 28% from 2000 to 2014 (1), and the mean paternal age also increased from 27.4 to 30.9 years over the past four decades (2). China Fertility Survey conducted in 2017 also showed that the mean age of women’s first birth increased by 2.6 years compared to that in 2006 (3). Evidence have shown that advanced parental age is associated with adverse reproductive outcomes, such as preterm birth (4), spontaneous miscarriage (5) or even stillbirth (6). But rarely do we lengthen our view beyond the live birth. Mounting studies have suggested that advanced paternal age may increase the risk of psychiatric disorders (6, 7) and telomere length (8) in offspring, and maternal age is strongly related to obesity (9), type 1 diabetes (10) and Down syndrome (11) in offspring. However, few studies have taken both maternal age and paternal age into consideration.

Elevated blood pressure (EBP) in children leads to an approximately two-fold increased risk of hypertension in adulthood (12), highlighting the importance to maintain a normal level of blood pressure in childhood. Recent research found that maternal factors and early life factors, such as birth weight, gestational age and breastfeeding (13–15), were related to offspring blood pressure levels. However, the relationship between parental age at delivery and children blood pressure remains under-studied. Moreover, previous studies showed that unhealthy lifestyle, including excess body weight (16), insufficient sleep duration (17), and unbalanced diet (18), could lead to hypertension in children. Whether healthy lifestyle could modify the associations between parental reproductive age and risk of offspring EBP is unknown.

In this study, we investigated the relationship of maternal and paternal age with risk of EBP among offspring, and further evaluated the role of childhood lifestyle in this relationship. Findings may add knowledge to children hypertension prevention in the future.

## Materials and methods

### Study design and participants

Data was obtained from the national school program (201202010) conducted in 7 Chinese provinces in 2013, including Shanghai, Guangdong, Hunan, Chongqing, Ningxia, Tianjin and Liaoning. The sampling procedure has been published previously (19). In brief, using random stratified cluster sampling method, 12–16 primary and secondary schools were selected in each province and 44,436 participants with complete information of maternal age and paternal age were included. In present study, we enrolled 39,190 children and adolescents aged 7–18 years after excluding the following participants: (a) children with missing values on weight (*n *= 427), height (*n *= 304), and blood pressure (*n *= 903); (b) children with extremely low birth weight (*n *= 1,910); (c) children who were not the singleton births (*n *= 1,702). The study was approved by the Ethics Committee of Peking University (No. IRB0000105213034). All participating students and their parents signed the informed consent.

### Measurements

All anthropometric and clinical measurements were conducted by qualified medical physicians from medical establishments. Height was measured without shoes using a portable stadiometer (model TZG, China) to the nearest 0.1 cm. Weight was measured in light clothing and without shoes using a lever-type weight scale (model RGT-140, China) to the nearest 0.1 kg. Body mass index (BMI) was then calculated as weight in kilograms divided by the square of height in meters (kg/m^2^). Blood pressure (BP) was obtained by Mercury sphygmomanometer (model XJ11D, China) and Stethophone (model TZ-1, China) from the right arm using the appropriate cuffs. Prior to the first reading, children were asked to seat quietly for at least 5 minutes. Systolic blood pressure (SBP) was defined as the onset of the first Korotkoff sound, and diastolic blood pressure (DBP) was defined as the fifth Korotkoff sound. BP was measured twice at a single visit and the averages of two readings for SBP or DBP were calculated for each participant.

### Covariates

Two sets of questionnaires were designed and applied for students and their parents, respectively. Children above fourth grade answered the student questionnaire independently, while students in grade 1–3 completed the questionnaires with the help of their parents. Children lifestyle information (e.g., diet behaviors, sleep duration and physical activities) were obtained from student questionnaires, whereas obstetric information (e.g., gestational age, delivery mode and birth weight), parental highest education level and family history of hypertension were obtained from parental questionnaires. Children were asked about daily consumption of fruits, vegetables, sugar-sweetened beverages and meat during the past 7 days, involving self-reported frequency (days) and amount (servings per day). Self-reported frequency was collected by asking “How many days in the past 7 days have you eaten fruit/vegetable/meat/beverage?”, and daily amount was collected by asking “On a fruit/vegetable/meat/beverage-eating day, how many servings did you eat per day on average?” and providing the 1 serving of reference, for example, 1 serving of fruit/vegetable is the size of an adult fist and 1 serving of meat is the size of the palm of your hand. Physical activity level was derived from children’ self-reported frequency (days) and duration (minutes per day) of moderate and vigorous-intensity activity over the past 7 days, following on the Chinese guidelines for data processing and analysis concerning the International Physical Activity Questionnaire (20). Sleep duration (h) was obtained by using the question “How long do you sleep every day?”. Both father’s and mother’s education degrees were collected, and the higher one was defined as the parental highest education degree.

### Definitions

Maternal/paternal age refers to the mother/father’s age when child was born, which was calculated by the differences of birth date between child and mother/father. Elevated blood pressure (EBP) was defined as blood pressure ≥90th percentile based on the guideline endorsed by American Academy of Pediatrics in 2017 (21). Based on Life’s essential 8 recommended by American Heart Association, BMI (22), physical activity (23), sleep (24) and diet (25) were used to evaluate the level of an individual’s lifestyle behavior for cardiovascular health. Thus, we intergrated these four factors to represent offspring overall lifestyle status, and their definitions and corresponding criteria were shown in Table 1. These factors were selected based on the evidence for their association with EBP and recommendations for BP management. Each factor was assigned a value of 1 if children meet the ideal standard, and the sum of total values was used as offspring lifestyle score. The offspring lifestyle score ranged from 0 to 4, with the higher score indicating a healthier lifestyle, and were further categorized as unfavorable lifestyle groups (those who scored 0–1) and favorable lifestyle group (those who scored 2–4).

**Table 1:** Definitions of healthy lifestyle factors.

Lifestyle factors	Definitions of healthy lifestyle factors
BMI groups	Overweight was defined as age- and sex-specific BMI z scores >1
	Thinness was defined as age- and sex-specific BMI z scores <−2
	Ideal BMI was defined as neither thinness nor overweight
Physical activities	Ideal physical activity was defined as ≥60 min of moderate or vigorous physical activities per day
Sleep duration	For children of 6–13 years: ≥9 and ≤11 h
	For teenagers of 14–17 years: ≥8 and ≤10 h
	Ideal sleep duration was defined as neither insufficient sleep nor hypersomnia
Dietary behaviors	Ideal dietary behavior was defined as satisfying 3–4 of following components:
	1) Vegetables: ≥4 servings per day^a^
	2) Fruits: ≥3 servings per day^a^
	3) Sugar-sweetened beverages:0 serving per day
	4) Meat consumption: ≥2 servings & ≤3 servings per day^b^

BMI, body mass index.

^a^ 1 serving = 100 g in raw material.

^b^ 1 serving = 50 g in raw material.

### Statistical analysis

Continuous variables were expressed as mean ± standard deviation and categorical variables were expressed as frequency (percentage). Restricted cubic spline (RCS) was used to estimate the nonlinear relationship between maternal age (paternal age) and the risk of EBP in offspring with 5 knots at the 10th, 27.5th, 50th, 72.5th and 95th. According to the interval of the lowest point for maternal age and the intersection of the curve for paternal age, we divided the sample into three maternal age groups (≤26, 26–32 and >32) and three paternal age groups (≤27, 27–30 and >30) respectively. A new variable with 9 categories was generated by integrating maternal age groups with paternal age groups. Logistic regression models were used to investigate the association between parental reproductive age and risk of EBP in offspring. Furthermore, we also carried out stratified analysis by offspring lifestyle groups. All analyses were performed by SPSS 25.0 (IBM SPSS Statistics 25.0, USA) and R studio 1.2.5033, and two-sided with *P* < 0.05 were considered as significant.

## Results

Of the final 39,190 participants enrolled in our study, 26.7% of them were defined as EBP (Table 2). Maternal age ranged from 18 years to 34 years, while paternal age ranged from 19 years to 37 years. The mean maternal and paternal age of the EBP group were 25.1 ± 3.5 years and 26.9 ± 3.8 years respectively, which were younger than those of the normotensives group. Compared with participants in the EBP group, children in normotensives group were more likely to had favorable lifestyle, none parental history of hypertension and educated parents (*P *< 0.001).

**Table 2:** Characteristics of participants involved according to offspring’s BP levels (*N* = 39,190).

	Normotensive	EBP	*P*-value
Number of children	28,735 (73.3)	10,455 (26.7)	
Age, y	10.5 ± 3.2	11.0 ± 3.3	<0.001
Sex			<0.001
Boy	13,651 (69.2)	6,070 (30.8)	
Girl	15,084 (77.5)	4,385 (22.5)	
Breastfeeding			0.001
Yes	24,325 (85.2)	8,969 (86.6)	
No	4,214 (14.8)	1,390 (13.4)	
Birth weight, g	3,328.6 ± 483.7	3,345.3 ± 489.2	0.10
Gestational age, week	39.7 ± 1.2	39.8 ± 1.1	<0.001
Delivery mode			0.71
Spontaneous delivery	16,442 (57.8)	5,941 (57.3)	
Caesarean section	12,009 (42.2)	4,420 (42.7)	
Lifestyle factors			
BMI groups			<0.001
Ideal	20,815 (72.4)	6,577 (62.9)	
Unideal	7,920 (27.6)	3,878 (37.1)	
Physical activities			<0.001
Ideal	7,796 (30.6)	3,062 (32.8)	
Unideal	17,706 (69.4)	6,269 (67.2)	
Sleep duration			0.40
Ideal	9,121 (34.8)	3,402 (35.3)	
Unideal	17,106 (65.2)	6,246 (64.7)	
Dietary behaviors			0.39
Ideal	51 (0.2)	23 (0.2)	
Unideal	26,534 (99.8)	9,633 (99.8)	
Lifestyle groups			<0.001
Unfavorable lifestyle	13,571 (57.8)	5,174 (60.1)	
Favorable lifestyle	9,897 (42.2)	3,441 (39.9)	
Maternal age, y	25.5 ± 3.4	25.1 ± 3.5	<0.001
Maternal age			<0.001
≤26	18,162 (63.6)	7,118 (68.6)	
26–32	9,338 (32.7)	2,817 (27.1)	
>32	1,076 (3.8)	448 (4.3)	
Paternal age, y	27.6 ± 3.8	26.9 ± 3.8	<0.001
Paternal age			<0.001
≤27	14,954 (52.2)	6,210 (59.7)	
27–30	6,881 (24.0)	2,229 (21.4)	
>30	6,812 (23.8)	1,964 (18.9)	
Parental history of hypertension			<0.001
None	25,994 (93.9)	9,376 (92.4)	
Yes	1,696 (6.1)	769 (7.6)	
Parental highest education degree			<0.001
Illiteracy or elementary school	790 (2.8)	345 (3.3)	
Junior high school	8,488 (29.8)	3,895 (37.5)	
Senior high school	7,941 (27.9)	2,973 (28.7)	
Technical secondary school/Junior college	5,309 (18.6)	1,664 (16.0)	
Undergraduate or above	5,955 (20.9)	1,496 (14.4)	

BP, blood pressure; EBP, elevated blood pressure; BMI, body mass index.

Continuous variables were expressed by mean values ± standard deviations, and categorical variables were expressed by frequency value (percentages, %).

**Figure 1:**
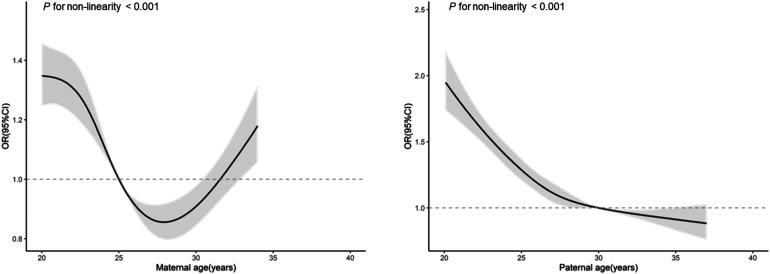
Parental reproductive age and risk of EBP in offspring.

Figure 1 depicts that as maternal age increased, the risk of EBP in offspring fell at first, reaching the lowest at around 26 to 32 years old, then rose with increasing maternal age. With the increase of paternal age, risk of EBP in offspring decline continuously, with the point of inflection at around 27 to 30 years. Based on these relationship characteristics, we applied corresponding cut-offs to categorize maternal age into ≤26, 26–32 and >32, and paternal age into ≤27, 27–30 and >30.

Table 3 (corresponding frequencies was shown in Supplementary Table S1) shows the relationship between maternal age and paternal age with the risk of EBP. In total, a U-shaped pattern was observed for maternal age and EBP, while the risk of EBP decreased constantly with the increase of paternal age (Model1). The associations changed slightly with further adjustment for confounding factors. Separately, participants who had the youngest (paternal age ≤27 years & maternal age ≤26 years) or the oldest (paternal age >30 years & maternal age >32 years) parental age groups were related to a greater risk of offspring EBP, compared with the combination of paternal age in 27–30 years and maternal age in 26–32 years, with ORs of 1.18 (95% CI: 1.08–1.29, *P *< 0.05) and 1.18 (95% CI: 1.01–1.38, *P *< 0.05), respectively. Considering the potential trend in different maternal age group, when maternal age was under 26 years, risk of EBP showed a downward trend with the increasing paternal age (*P*_trend _< 0.001). As for trends in distinct paternal age group, risk of EBP seemed to decrease with maternal age when paternal age was below 27 years (*P*_trend _< 0.001), but increase with maternal age when paternal age was older than 30 years (*P*_trend _= 0.02). The interaction between maternal/paternal age groups and lifestyle factors were also tested, though it was not statistically significant.

**Table 3:** Relationship between parental reproductive age and risk of EBP in offspring [OR (95% CI)].

Paternal age	Model 1				Total	Model 2				
	Maternal age			*P*_trend_		Maternal age			*P*_trend_	Total
	≤26	26–32	>32			≤26	26–32	>32		
≤27	**1.29** (1.19–1.39)* **	**1.00** (0.89–1.12)	**0.97** (0.41–2.07)	<0.001	1.27 (1.20–1.34)***	**1.18** (1.08–1.29)***	**0.96** (0.83–1.10)	**1.18** (0.46–2.75)	<0.001	1.18 (1.10–1.26)***
27–30	**0.98** (0.89–1.08)	1.00 (ref)	**1.12** (0.70–1.75)	0.64	1.00 (ref)	**0.96** (0.86–1.07)	1.00 (ref)	**0.96** (0.55–1.62)	0.39	1.00 (ref)
>30	**0.77** (0.68–0.88)* **	**0.86** (0.78–0.94)**	**1.35** (1.18–1.54)***	<0.001	0.91 (0.85–0.98)*	**0.84** (0.73–0.97)*	**0.88** (0.79–0.99)*	**1.18** (1.01–1.38)*	0.02	0.93 (0.86–1.02)
*P*_trend_	<0.001	0.002	0.29			<0.001	0.21	0.62		
Total	1.27 (1.21–1.33)***	1.00 (ref)	1.42 (1.26–1.59)***			1.18 (1.11–1.25)***	1.00 (ref)	1.23 (1.07–1.41)**		

Model 1 adjusted for offspring age and sex.

Model 2 adjusted for offspring age, sex, province, school, breastfeeding, birth weight, gestational age, delivery mode, parental history of hypertension, parental highest education degree and offspring lifestyle score.

* *P *< 0.05, **P < 0.01, ***P < 0.001.

**Figure 2:**
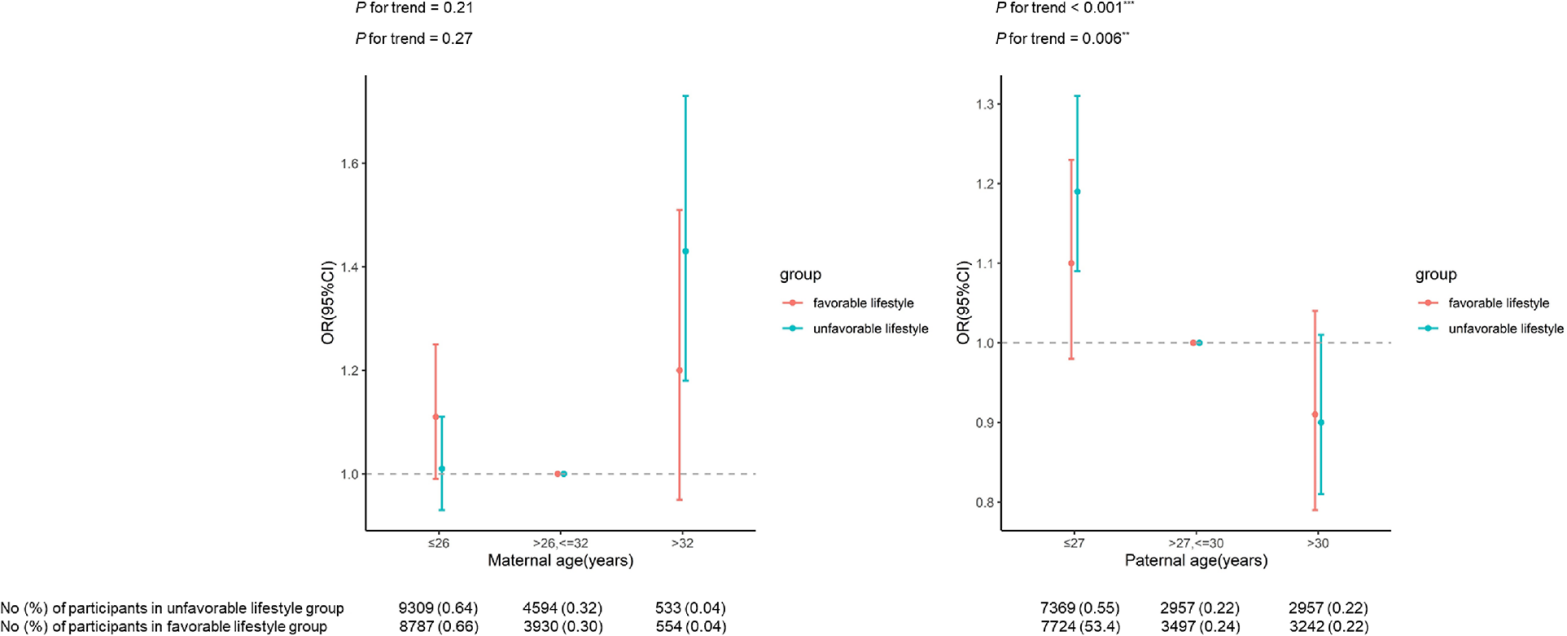
Relationship between parental reproductive age and the risk of EBP by offspring lifestyle groups.

Figure 2 presents the association between maternal/paternal age and risk of EBP in different lifestyle groups. In unfavorable lifestyle group, an increased risk of EBP was found when maternal age was >32 years (OR = 1.43, 95% CI: 1.18–1.73) and paternal age was ≤27 years (OR = 1.19, 95% CI: 1.09–1.31), after adjusted for the reproductive age of spouse and other covariates. However, these relationships were not found in participants of favorable lifestyle group. Results were not different when stratified by offspring sex (Supplementary Table S2–S3), though these patterns were not found for any single lifestyle factor (Supplementary Table S4–S7).

## Discussion

Our findings observed a U-shaped pattern for maternal age and EBP in offspring, but a continuing downward trend for paternal age under 37 years. Combining maternal age and paternal age, children born to younger and older parents have higher risk of EBP compared with those of moderate age parents. Furthermore, stratified analysis showed that the association between parental reproductive age and risk of EBP was not found in children with a healthier lifestyle, suggesting that offspring healthy lifestyle could mitigate the adverse effect of parental age on their BP profile.

Previous studies showed inconsistent results regarding the influence of parental age on the risk of EBP. Several studies demonstrated that higher maternal age was associated with higher SBP in newborn or young children (26–28), but these studies did not take paternal age into the consideration. When both maternal age and paternal age were taken into account, David and colleagues (29) found that advanced maternal age and paternal age, separately, could lead to higher blood pressure in offspring at 18 years. Though the influence of paternal age was strong, it was attenuated by adjustment of maternal age. Another study conducted in New Zealand (30) using mean parental age at childbirth (MPAC) showed that increasing MPAC was related to reductions in night-time SBP and DBP. However, that study failed to distinguish the effect of maternal age and paternal age separately. Moreover, Leary (31) using the data on 4,723 parent-child pairs did not find any influence of maternal age and paternal age on offspring blood pressure. Our study extended the evidence from previous findings, which not only examined the independent association of maternal age and paternal age with offspring EBP, but also evaluated the joint effect of maternal and paternal age by applying a parental age group with 9 categories. Furthermore, we described the U-shaped pattern for maternal age and continuing downward trend for paternal age, which was corroborated both in total and different age stratums.

Our findings indicated that both young and old maternal age could increase the risk of EBP in offspring. Florent (32) found a U-shaped pattern between maternal age and preterm birth, which could be a possible explanation for our findings because infants who were born preterm tend to have high systolic blood pressure later in life (33). Studies have signified the role of aging oocytes which present with oocyte cytoplasmic volume decreasing (34) and mitochondrial dysfunction (35), as well as epigenetic modifications occurred in genes regulating growth and metabolism, such as histone acetylation and DNA methylation (36). Similar epigenetic changes with increasing paternal age (37–39) may also be associated with changes of metabolic phenotype in offspring, but the specific mechanism remained unclear. In addition, the protective effect of older paternal age we found is possibly because that older fathers are more likely to be wealthier and better educated compared with younger fathers, which enable them to provide a better and healthier child-bearing environment for children (40, 41). Although we have adjusted for parental highest educational degree in this analyses, the household income was not taken into account due to the insufficient information collected.

Although existing studies have separately demonstrated the effect of diet, physical activity, weight loss and sleep on children blood pressure, it is necessary to integrate these lifestyle factors to evaluate the overall lifestyle status of children. In this study, we demonstrated that children maintaining a healthier lifestyle could contribute to offset the detrimental influence of parental age on offspring blood pressure, providing new insights to promote healthy BP during childhood. Consistent with our findings, Simonetta (42) also demonstrated the significant efficacy of lifestyles modification on children blood pressure. These results were also supported by another Chinese study (43), which reported that the risk of offspring metabolic syndrome was lower in those who had more ideal lifestyle factors. Moreover, given that there is still a *p* for trend in paternal age in the favourable group, conclusion regarding the offset effect of lifestyle on association of paternal age should be drawn with caution.

In general, from a public health perspective, our study highlights the adverse effect of both young and advanced parental age on risk of EBP in children. Furthermore, the present study also suggests that adherence to healthy lifestyles could possibly offset the adverse effect of parental reproductive age on offspring blood pressure. These findings may be particularly important considering that the three-child policy was recently released by the Chinese government, which may lead to an increase in number of children with older parental age.

Several limitations should be considered when our findings were interpreted. First, causality is difficult to infer from this observational study. Second, BP measurements were taken in one visit for each individual, which may overestimated the prevalence of EBP. Third, there was not enough sample of older parents and the conclusion only limited to the age range in present study. Fourth, instrumental measurements should be used to obtain more accurate and objective results rather than questionnaires, such as actigraph for monitoring energy consumption. Finally, pre-pregnancy maternal factors and puberty development of children were not considered in this study, which could be potential confounders. Information regarding some diseases that may influence blood pressure should also be collected to exclude unqualified participants.

In conclusion, our study found that children born to young or old parents had greater risk for EBP. However, healthy childhood lifestyles could mitigate the adverse effect of parental age on children EBP. Our findings highlight the role of parental reproductive age on offspring EBP, and the importance of maintaining healthy lifestyle for children, which may aid current prevention strategy aiming to reduce risk of hypertension in the future.

## Data Availability

The original contributions presented in the study are included in the article/**Supplementary Material**, further inquiries can be directed to the corresponding author.
