# Supplementary material for: Associations of parental reproductive age and elevated blood pressure in offspring: An observational study
Source: Front Pediatr. 2023 Mar 30;11:990725. doi: 10.3389/fped.2023.990725 (PMC10098010; doi:10.3389/fped.2023.990725)
Supplement: Supplementary file 1 [file Datasheet1.pdf]

TableS1 Frequencies (%) of parental reproductive age

Paternal age (y)	Maternal age (y)		
	≤26	26~32	>32
≤27	18730 (48.2)	2217 (5.7)	34 (0.1)
27~30	4400 (11.3)	4598 (11.8)	97 (0.2)
>30	2035 (5.2)	5338 (13.7)	1392 (3.6)

TableS2 Relationship between parental reproductive age and the risk of EBP by offspring lifestyle in **boys** [OR (95%CI)]

Offspring lifestyle	Maternal age (y)			P_{trend}	Paternal age (y)			P_{trend}
	≤ 26	26 ~ 32	> 32		≤ 27	27 ~ 30	> 30	
Unfavorable	1.05 (0.93~1.18)	1.00 (ref)	1.29 (0.98~1.69)	0.96	1.14 (1.01~1.30)*	1.00 (ref)	0.97 (0.84~1.13)	0.02
Favorable lifestyle	1.02 (0.88~1.19)	1.00 (ref)	1.03 (0.75~1.39)	0.88	1.07 (0.91~1.24)	1.00 (ref)	0.95 (0.79~1.13)	0.19

Note: Adjusted for paternal age (maternal age), offspring age, sex, province, school, breastfeeding, birth weight, gestational age, delivery mode, parental history of high blood pressure, parental highest education degree.

TableS3 Relationship between parental reproductive age and the risk of EBP by offspring lifestyle in **girls** [OR (95%CI)]

Offspring lifestyle	Maternal age (y)			P_{trend}	Paternal age (y)			P_{trend}
	≤ 26	26 ~ 32	> 32		≤ 27	27 ~ 30	> 30	
Unfavorable lifestyle	0.97 (0.85~1.11)	1.00 (ref)	1.60 (1.21~2.09)***	0.05	1.27 (1.10~1.46)***	1.00 (ref)	0.83 (0.70~0.98)*	<0.001
Favorable lifestyle	1.26 (1.06~1.49)**	1.00 (ref)	1.45 (1.02~2.02)*	0.15	1.16 (0.97~1.38)	1.00 (ref)	0.89 (0.72~1.10)	0.01

Note: Adjusted for paternal age (maternal age), offspring age, sex, province, school, breastfeeding, birth weight, gestational age, delivery mode, parental history of high blood pressure, parental highest education degree.

$^{*}P<0.05$, $^{**}P<0.01$, $^{***}P<0.001$

TableS4 Relationship between parental reproductive age and the risk of EBP by **children BMI groups** [OR (95%CI)]

BMI groups	Maternal age (y)			P trend	Paternal age (y)			P trend
	≤ 26	26 ~ 32	> 32		≤ 27	27 ~ 30	> 30	
Unideal	1.07 (0.96~1.19)	1.00 (ref)	1.35 (1.06~1.70)*	0.90	1.13 (1.01~1.26)*	1.00 (ref)	0.88 (0.77~0.99)*	<0.001
Ideal	1.03 (0.96~1.12)	1.00 (ref)	1.32 (1.12~1.55)***	0.53	1.16 (1.07~1.26)***	1.00 (ref)	0.88 (0.80~0.97)*	<0.001

Note: Adjusted for paternal age (maternal age), offspring age, sex, province, school, breastfeeding, birth weight, gestational age, delivery mode, parental history of high blood pressure, parental highest education degree.

*P<0.05, **P <0.01, ***P <0.001

TableS5 Relationship between parental reproductive age and the risk of EBP by **children physical activities** [OR (95%CI)]

Physical activities	Maternal age (y)			P trend	Paternal age (y)			P trend
	≤ 26	26 ~ 32	> 32		≤ 27	27 ~ 30	> 30	
Unideal	1.06 (0.98~1.15)	1.00 (ref)	1.41 (1.19~1.67)***	0.81	1.14 (1.05~1.24)**	1.00 (ref)	0.91 (0.82~0.99)*	<0.001
Ideal	1.02 (0.90~1.15)	1.00 (ref)	1.19 (0.93~1.53)	0.73	1.19 (1.05~1.34)**	1.00 (ref)	0.88 (0.76~1.03)	<0.001

Note: Adjusted for paternal age (maternal age), offspring age, sex, province, school, breastfeeding, birth weight, gestational age, delivery mode, parental history of high blood pressure, parental highest education degree.

*P<0.05, **P <0.01, ***P <0.001

TableS6 Relationship between parental reproductive age and the risk of EBP by **children sleep duration** [OR (95%CI)]

Sleep duration	Maternal age (y)			P_{trend}	Paternal age (y)			P_{trend}
	≤ 26	26 ~ 32	> 32		≤ 27	27 ~ 30	> 30	
Unideal	1.02 (0.94~1.10)	1.00 (ref)	1.33 (1.11~1.57)**	0.33	1.19 (1.09~1.30)***	1.00 (ref)	0.87 (0.79~0.96)**	<0.001
Ideal	1.18 (1.06~1.33)**	1.00 (ref)	1.29 (1.02~1.62)*	0.08	1.08 (0.96~1.21)	1.00 (ref)	0.96 (0.84~1.11)	0.09

Note: Adjusted for paternal age (maternal age), offspring age, sex, province, school, breastfeeding, birth weight, gestational age, delivery mode, parental history of high blood pressure, parental highest education degree.

*P<0.05, **P <0.01, ***P <0.001

TableS7 Relationship between parental reproductive age and the risk of EBP by **children dietary behaviors** [OR (95%CI)]

Dietary behaviors	Maternal age (y)			P trend	Paternal age (y)			P trend
	≤ 26	26 ~ 32	> 32		≤ 27	27 ~ 30	> 30	
Unideal	1.06 (0.99~1.13)	1.00 (ref)	1.32 (1.15~1.52)***	0.90	1.16 (1.08~1.24)***	1.00 (ref)	0.89 (0.82~0.97)**	<0.001
Ideal	1.28 (0.20~1.02)	1.00 (ref)	—	0.96	1.33 (0.24~8.62)	1.00 (ref)	—	0.26

Note: Adjusted for paternal age (maternal age), offspring age, sex, province, school, breastfeeding, birth weight, gestational age, delivery mode, parental history of high blood pressure, parental highest education degree.

"—" The frequency of this cell was too small to estimate.

**P <0.01, ***P <0.001
